# The neural circuits of innate fear: detection, integration, action, and memorization

**DOI:** 10.1101/lm.042812.116

**Published:** 2016-10

**Authors:** Bianca A. Silva, Cornelius T. Gross, Johannes Gräff

**Affiliations:** 1 Laboratory of Neuroepigenetics, Brain Mind Institute, Faculty of Life Sciences, Ecole Polytechnique Fédérale Lausanne, CH-1015 Lausanne, Switzerland; 2 Mouse Biology Unit, European Molecular Biology Laboratory (EMBL), 00015 Monterotondo, Italy

## Abstract

How fear is represented in the brain has generated a lot of research attention, not only because fear increases the chances for survival when appropriately expressed but also because it can lead to anxiety and stress-related disorders when inadequately processed. In this review, we summarize recent progress in the understanding of the neural circuits processing innate fear in rodents. We propose that these circuits are contained within three main functional units in the brain: a detection unit, responsible for gathering sensory information signaling the presence of a threat; an integration unit, responsible for incorporating the various sensory information and recruiting downstream effectors; and an output unit, in charge of initiating appropriate bodily and behavioral responses to the threatful stimulus. In parallel, the experience of innate fear also instructs a learning process leading to the memorization of the fearful event. Interestingly, while the detection, integration, and output units processing acute fear responses to different threats tend to be harbored in distinct brain circuits, memory encoding of these threats seems to rely on a shared learning system.

The term “fear” refers to a human emotion characterized by the conscious feeling of being afraid, and as such it is not clear whether a similar emotion also occurs in other species (Panksepp 1989; LeDoux 2012; Anderson and Adolphs 2014). In the field of neuroscience, “fear” is also used to refer to the collective defensive responses elicited by dangers across species and sometimes also to the neural systems that mediate these responses. We favor a more general definition that refers to “fear” as a central state, which is induced when the subject perceives danger and that mediates bodily and behavioral responses to such danger (Adolphs 2013). These responses include defense mechanisms that are necessary for the survival of the individual and can be observed in virtually all animal species. Fear responses are triggered by a variety of stimuli, including predators, aggressive members of the same species, pain, and dangerous features of the environment such as heights. Importantly, these types of stimuli strongly and systematically induce defensive behaviors and do not depend on the experience of direct harm associated with the threat nor on a learning process assigning a valence of danger to the threat. This type of fear is what has been referred to as “innate fear” (Blanchard and Blanchard 1989).

Nevertheless, an innate fear-inducing experience, besides producing an acute adaptive response, also induces the formation of a memory of the fearful event. This is mediated by long-lasting changes in the brain and is aimed to decrease the possibility to reencountering the same threat and to better cope with similar future events. One component of this memory is the association between the innate fear-inducing stimulus and a neutral stimulus such as, for example, the context where it was encountered. This associative form of memory, where a neutral stimulus acquires the ability to induce defensive responses, has been referred to as “conditioned or learned fear” and has for long been the main focus of research attempts to unravel the neural basis of fear (LeDoux 2003, 2014). Accordingly, the brain circuits (for recent reviews, see Herry and Johansen 2014; Tovote et al. 2015), as well as the genetic and molecular underpinnings thereof have been described in great detail (Rodrigues et al. 2004; Johansen et al. 2011) and will not be repeated here. Importantly, innate and conditioned fear responses seem to be mediated, at least partially, by nonoverlapping circuits (Gross and Canteras 2012), making it impossible to simply transfer our exhaustive understanding of the neural processing of conditioned fear to innate fear.

In this review, we summarize the current knowledge on the brain circuits processing innate fear responses to a wide variety of threats, including predators, aggressive conspecifics and painful stimuli. Accumulating evidence indicates that innate fear to these different types of threats relies on parallel nonoverlapping circuits. Nevertheless, these circuits seem to share a common organization into three fundamental functional levels (Fig. 1): a detection unit composed of different sensory systems, including vision, olfaction, audition, and nociception; an integration unit where the different types of sensory information converge to recruit downstream structures initiating adaptive responses; and an output unit composed of structures directly initiating behavioral and bodily responses. Furthermore, experiencing innate fear can lead to a memorization of the event and thus represents an essential condition for fear learning. In light of this, we also elaborate on the interactions between neural circuits processing innate and learned fear with a particular focus on how innate fear signals instruct fear memorization (Fig. 2).

**Figure 1.**
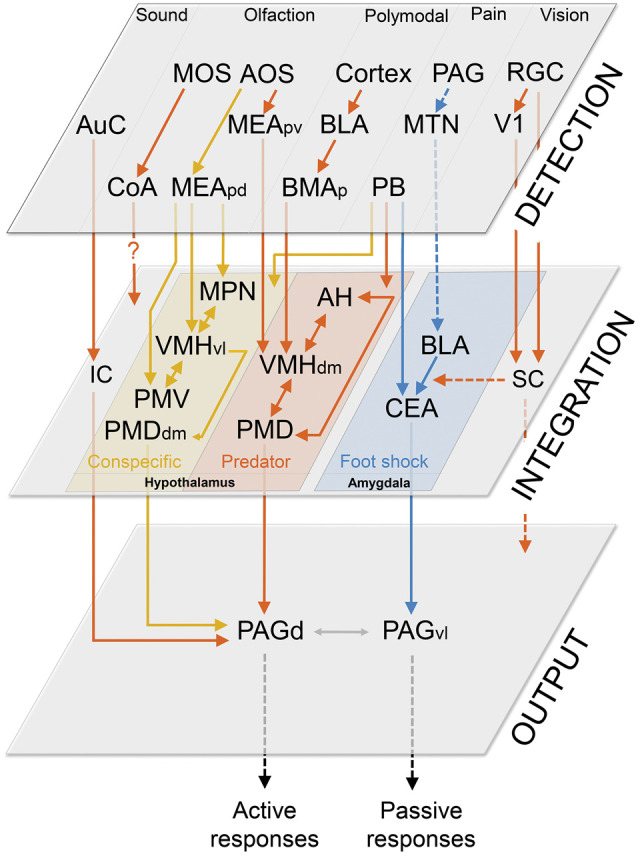
Schematic representation of the neural circuits mediating innate fear to different threats. Three main functional units process innate fear, a detection unit (*upper* plane), an integration unit (*middle* plane), and an output unit (*lower* plane). Information about the threat is collected through different sensory modalities. Acoustic inputs (such as ultrasounds) are processed by the auditory cortex (AuC), which in turn projects to the inferior colliculus (IC) that sends afferents to the dorsal periaqueductal gray (PAGd). Moving visual stimuli in the upper visual field are processed by the superior colliculus (SC), which receives inputs from the retinal ganglion cells (RGN) and primary visual cortex (V1) and mediates fear responses through targeting the amygdala and brainstem. Olfaction plays a crucial role in the detection of both predator (orange) and conspecific (yellow) signals. The main olfactory system (MOS) mediates defensive responses to the predator odor via projections to the cortical amygdala (CoA), but the outputs of this structure mediating behavioral responses remain unclear. The accessory olfactory system (AOS) signals conspecific cues to the posterior dorsal portion of the medial amygdala (MEAdd) and predator cues to its posterior ventral portion (MEApv). These two medial amygdalar nuclei project to the conspecific and predator integration circuits in the hypothalamus. The predator fear circuit also receives polymodal sensory information about the threat via a basolateral amygdala (BLA)-basomedial amygdala (BMA) circuit. The hypothalamic integration unit processing conspecific fear includes four highly interconnected nuclei: the medial preoptic nucleus (MPN), the ventrolateral portion of the ventromedial hypothalamic nucleus (VMHvl), ventral premammillary nucleus (PMV), and dorsomedial portion of the dorsal premammillary nucleus (PMDdm). The conspecific fear circuit mediates defensive responses through its projections to the PAGd. The predator fear circuit consists of the anterior hypothalamic nucleus (AH), the VMHdm, and the PMD and mediates defensive responses through projections to the PAGd. Importantly, both the conspecific and predator hypothalamic circuits receive nociceptive information from the parabrachial nucleus (PB). Defense to painful stimuli (blue lines) such as an electrical footshock is mediated by activation of the ventrolateral periaqueductal gray (vlPAG) via the central nucleus of the amygdala (CEA). The CEA receives noxious information from the parabrachial nucleus (PB). The basolateral amygdala complex (BLA) plays a major role in footshock-induced fear through its projections to the CEA. The BLA integrates nociceptive information from the PAG via midline thalamic nuclei (MTN).

**Figure 2.**
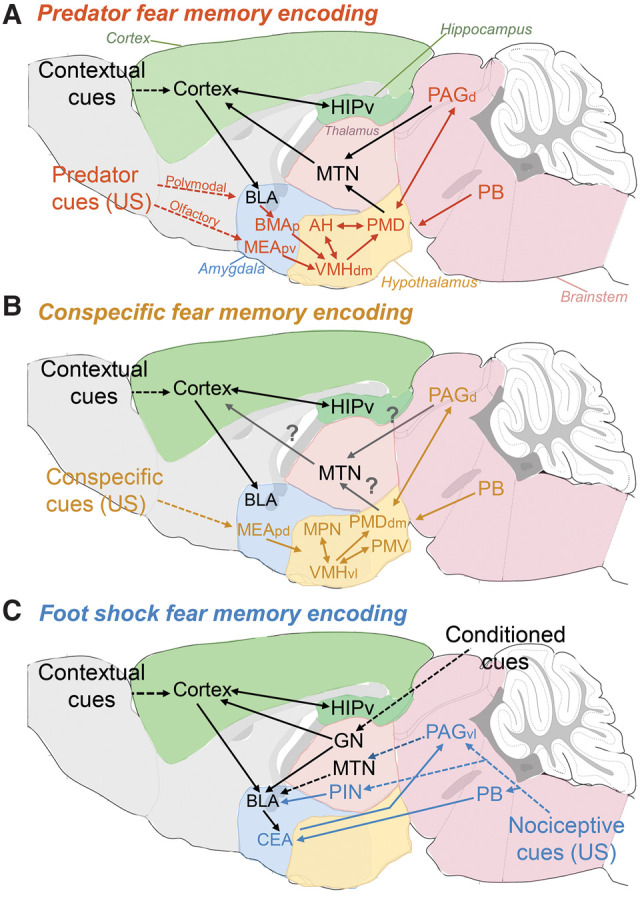
Hypothetical circuits mediating memorization of fear to different classes of threats. Innate fear circuits for all classes of threats interact with a common memorization unit centered in the amygdala, hippocampus, and cortex (black lines). (*A*) Memorization of predator fear. Information about the predator (US inputs: orange lines) are conveyed to the hypothalamus through the amygdala with the MEApv processing olfactory information and the posterior portion of the basomedial amygdala (BMAp) processing cortical polymodal information conveyed through the basolateral amygdala (BLA). In addition, the MHDS receives afferents from the parabrachial nucleus (PB), which probably conveys pain signals that may emerge during the encounter with a predator. The medial hypothalamic defensive system (MHDS), composed of the anterior nucleus (AH), the dorsomedial portion of the ventromedial hypothalamus (VMHdm), and the dorsal premammillary nucleus (PMD), integrates sensory-derived information and drives acute defensive responses through its downstream projections to the dorsal portion of the priaqueductal gray (PAGd). The MHDS and the PAGd send upstream projections to midline thalamic nuclei (MTN), including the anteromedial thalamic nucleus which, in turn, projects to cortical structures including the anterior cingulate, retrosplenial, entorhinal, and perirhinal cortices, areas implicated in contextual fear learning. These upstream projections from hypothalamic and PAG innate fear circuit may convey US signals to the memorization unit involving cortical, amygdalar, and hippocampal structures. The precise processing of CS–US integration remains unclear. (*B*) Memorization of conspecific fear. This circuit has not been fully investigated; as a result, this part of the model is highly speculative. Information about the aggressive conspecific (US inputs: yellow lines) is conveyed to the hypothalamus through the MEApd. The hypothalamic circuit involving the medial preoptic nucleus (MPO), the ventrolateral portion of the ventromedial hypothalamus (VMHvl), the ventral premammillary nucleus (PMV) and the dorsomedial portion of the dorsal premammillary nucleus (PMDdm) integrate conspecific signals and drive acute defensive responses through its downstream projections to the PAGd. Similarly to the predator system PB inputs to these hypothalamic nuclei might convey nociceptive information that emerge in the case of attacks by an aggressive conspecific. It is possible that the conspecific innate fear circuit sends upstream projection to midline thalamic nuclei and thereby instructs memory formation similarly to the predator fear circuit. However, this has not been directly addressed. (*C*) Memorization of footshock fear. Contextual information (CS) are conveyed through cortico-thalamic inputs to the basolateral amygdalar complex (BLA) and ventral hippocampus (HIPv), while other conditioned cues such as auditory inputs are conveyed to the BLA and cortex through sensory thalamic nuclei such as the geniculate nuclei (GN). How nociceptive information (US inputs: blue lines) reaches the cortico-amygdalo-hippocampal memorization system is less clear. A spino-thalamic tract may convey nociceptive inputs to the BLA and to other cortical structures through the posterior intralaminar thalamic nuclei (PIN). However, ibotenic lesions at this level do not impair fear learning. Another possible source of nociceptive inputs may arise from the ventrolateral periaquiductal grey (PAGvl) targeting the BLA through MTN relay stations. Moreover, the brainstem parabrachial nucleus (PB) directly conveys nociceptive inputs to the central amygdala (CEA), which, in turn, mediates defensive behaviors via its projections to the PAGvl. The implicated brain structures have been indicated at their approximate positions so as to still keep the legend readable and are colored for ease of reading.

It is important to note that our current understanding of innate fear circuits largely derives from studies based on predator fear, as other classes of threat, including aggressive conspecifics or physically harmful threats, have been less studied. Here, we attempt to comprehensively summarize the neural circuits recruited by exposure to all kinds of threat, bearing in mind that certain aspects can only be addressed in light of predator fear research.

## Detection unit

Although threats vary for different species, here we will focus on rodents as they are the most commonly used model organisms for the study of fear circuits.

Three main classes of threats inducing innate fear in rodents exist: predators, aggressive members of the same species, and internal information such as painful stimuli or suffocation signals. All these types of threats are detected by the brain via different sensory modalities, including olfaction, vision, audition, and nociception. Interestingly, many examples show that signals from a single sensory modality such as predator odor, a moving shadow from above, or ultrasound calls are independently sufficient to drive acute defensive responses.

### Olfactory threat cues

In contrast to humans, rodents mainly rely on their sense of smell to collect information about the environment. Most olfactory signals have the capability to instruct behavior through experiential association, yet a subset of odorants innately drives defensive responses (Stowers and Kuo 2015). These include odorants from bodily secretions like urine, feces, or saliva from predators including felines, rats, snakes, and predatory birds (Isogai et al. 2011), as well as odors from stressed conspecifics (Brechbühl et al. 2008; Stowers and Logan 2010; Rosen et al. 2015). A number of predator-derived single molecules capable of inducing innate fear have been identified, which include urinary protein homologues from cat fur (Papes et al. 2010), 2-phenylethylamine from the urine of carnivores (Ferrero et al. 2011), and 2,4,5-trimethylthiazoline (TMT) from fox feces (Rosen et al. 2015).

The detection of odorants signaling danger relies on two main systems: the main olfactory system (MOS) and the accessory olfactory system (AOS). The MOS is capable of sensing an extremely wide range of volatile molecules conveying information about the environment, whereas the AOS appears to serve a more specialized function for the detection of odorants from other individuals. In particular, the AOS senses pheromones (Dulac and Torello 2003), which signal intraspecific information and kairomones—that is, odorants derived from nonconspecific individuals such as predators (Ben-Shaul et al. 2010; Papes et al. 2010). Additionally, a third olfactory organ located in the nasal cavity, the Grueneberg ganglion cells system, relays olfactory signals of danger mainly derived from injured conspecifics (Brechbühl et al. 2008). Interestingly, all these olfactory systems can directly instruct fear responses to different predator odorants recruiting partially divergent downstream brain circuits (Takahashi 2014; Pérez-Gómez et al. 2015).

The MOS detects volatile odorants through sensory neurons of the olfactory epithelium that project to specific structures in the main olfactory bulb (MOB) called glomeruli, whereas the AOS detects fluid-phase chemicals through the vomeronasal organ (VNO), a chemoreceptive structure located at the base of the nasal septum that projects to the accessory olfactory bulb (AOB Meredith 1991; Breer et al. 2006; Isogai et al. 2011). Detailed insight into the function of the MOS in the innate fear circuit is derived from studies using the fox feces-derived molecule TMT as a predator signal (for a review, see Rosen et al. 2015). TMT is detected by nasal epithelium neurons projecting to mitral cells in the posterior dorsal olfactory bulb (Kobayakawa et al. 2007; Matsumoto et al. 2010), which, in turn, project to the anterior cortical amygdala (CoA, Miyamichi et al. 2011), a structure driving defensive behaviors (Root et al. 2014). Accordingly, optogenetic inhibition of the CoA reduces TMT-induced defensive behavior, whereas selective activation of TMT-responsive CoA neurons is sufficient to recapitulate TMT-induced fear behaviors (Root et al. 2014). However, the target structures of CoA neurons mediating defensive responses have not been identified. Yang et al. (2016) recently described a putative circuit processing TMT signals, where glutamatergic projections from the lateral habenula activate parvalbumin-positive (PV+) interneurons in the laterodorsal tegmentum, which in turn drive fear responses including freezing, accelerated heart rate, and increased serum corticosterone levels. Other areas may also contribute to the processing TMT-induced fear such as the bed nucleus of the stria terminalis (BNST; Fendt et al. 2003), the lateral septum (LS; Endres and Fendt 2008), and the central amydgala (CEA; Isosaka et al. 2015), as their inactivation reduces TMT-inducer fear behaviors. Lastly, a recent paper showed a specific area of the olfactory cortex, the amygdalo piriform transition area, to mediate stress hormone responses to predator odors (Kondoh et al. 2016). Yet, a comprehensive understanding of the interplay between the pathways processing TMT-induced fear responses remains to be determined.

Differently from the MOS, the AOS relies on a separate set of brain structures to convey threat signals. In particular, the AOB, which processes vomeronasal inputs, sends projections to the medial amygdala (MEA), a structure-mediating innate predator and conspecific fear (Motta et al. 2009) as evidenced by c-Fos mapping and lesion studies (Li et al. 2004; Blanchard et al. 2005; Motta et al. 2009). The MEA, in turn, serves as a major input to the medial hypothalamic defensive system (for detailed description see “integration unit”) that integrates olfactory and nonolfactory stimuli and recruits downstream structures like the dorsal PAG (PAGd) for the initiation of defensive behaviors (Canteras 2002).

The olfactory threat detection unit mainly serves the innate fear system to sense the presence of predators. Nevertheless, fear responses are also elicited by exposure to aggressive conspecifics and olfaction seems to play an important role to signal social threats. For example, the VNO is recruited by exposure to a number of intraspecific signals including androgens (Isogai et al. 2011) found in the sweat of males of most mammalian species and able to induce elevated stress hormones and stress-mediated analgesia in mice (Sorge et al. 2014). In addition, odorants from stressed or injured conspecifics can also signal danger. Interestingly, these signals are detected by the Grueneberg ganglion cells system (Brechbühl et al. 2008). Grueneberg ganglion neurons are located in the anterior region of the nasal cavity and project to the MOB, but the downstream structures recruited by these neurons in the modulation of defensive responses have not been identified.

### Visual threat cues

Mice and rats display defensive responses including flight, shelter seeking, and freezing to looming shadows in the upper visual field (Wallace et al. 2013; Yilmaz and Meister 2013). In the laboratory, these responses have been mimicked by displaying an expanding dark circle or a moving bar on a screen positioned on top of the animal's cage and are likely to reflect natural responses to approaching birds of prey. Indeed, vision is the only effective way to detect these types of threat. Interestingly, laboratory mice seem to show higher freezing when exposed to an overhead looming stimulus than to a predator odor (Apfelbach et al. 2005; Yilmaz and Meister 2013), which might reflect an adaptation of rodents to prevent strictly visually guided aerial predators from detecting them (Yilmaz and Meister 2013).

Despite the robustness of looming stimuli-evoked defensive behaviors, we still know very little about the neural processing underlying such responses. The very short latency of defensive responses to looming stimuli (down to 250 msec; Ylimaz and Meister 2013) suggests a limited amount of central processing and possible direct visual inputs to brainstem defense systems, at least for the immediate freezing and escape responses. Different lines of experimental evidence suggest the SC as a major regulator of visual stimuli-induced defensive behaviors. SC is activated by overhead shadows (Zhao et al. 2014; Shang et al. 2015), its electrical and chemical stimulation induce defensive behaviors (Sahibzada et al. 1986; Dean et al. 1988; Keay et al. 1988; Schenberg et al. 1990; Sudré et al. 1993), and SC lesions impair defensive reactions to an approaching experimenter and to a sudden overhead visual stimulus (Dean et al. 1989).

Several circuits centered in the SC have been proposed for the processing of looming stimuli-induced defensive responses. For instance, using optrode recordings and optogenetic manipulations, Shang et al. (2015) identified a retino-SC-parabigeminal nucleus circuit with PV+ excitatory projection neurons located in the SC serving as a sensory-motor processor of looming-induced stimuli. In contrast, Liang et al. (2015) showed that V1 layer 5 neurons projecting to the SC can directly influence defensive behavior to a visual stimulus, thus suggesting an alternative circuit for SC recruitment upon visual stimuli exposure (Liang et al. 2015). Yet another route of visual detection was identified by Wei et al. (2015) who showed that CaMKII positive cells in the SC responding to looming stimuli mediate defensive behaviors by recruiting the lateral amygdala (LA) via the lateral posterior nucleus of the thalamus. The precise hierarchical interactions between these circuits and their reciprocal connections are currently under investigation. One possible explanation of these apparently redundant circuits is that the retino-PV+SC-brainstem circuit mediates active escape responses associated with increased heart rate (Shang et al. 2015), whereas the CaMKII+ SC-lateral posterior thalamus-LA circuits mediates passive responses like freezing associated with bradycardia (Wei et al. 2015), likely via a CEA–ventrolateral periaqueductal gray (PAGvl) output circuit (see below).

### Auditory threat cues

Mammals frequently depend on their sense of hearing to detect threats, particularly in nocturnal species. As a result, auditory cues have widely been used to elicit defensive responses in associative learning paradigms where animals learn to associate an electrical footshock with a neutral sound. The neural circuits underlying such tone-induced conditioned fear responses have been investigated in considerable depth (Herry and Johansen 2014; Tovote et al. 2015). However, under certain conditions, auditory stimuli can themselves induce innate fear responses. For instance, 17–22 kHz ultrasound tones can induce flight and freezing responses in mice (Mongeau et al. 2003) probably recruiting a specialized threat-detection system, given that ultrasonic vocalizations serve as alarm cries in rats exposed to predator odor (Blanchard et al. 1991; Litvin et al. 2007). Only a few studies have investigated the neural circuits of sound-induced innate fear. Mongeau et al. (2003) showed that mice exposed to ultrasonic tones in their home cages show increased c-Fos expression in the inferior colliculus (IC) region responsive to 17–20 kHz sounds, and in the dorsal PAG. In line with these findings, Xiong et al. (2015) used pharmacological and optogenetic manipulations to identify an auditory cortex–IC–PAGd circuit mediating flight responses to 1–64 kHz loud noise. Yet, other circuits are likely to be identified in future studies.

### Noxious stimuli

Acute fear responses to pure noxious stimuli have not been investigated in depth and it is currently not clear whether these stimuli are indeed sufficient to induce innate fear responses (Box 1). In contrast, painful stimuli like an electrical foot shock have widely been used in the study of conditioned fear where animals learn to associate the noxious aversive stimulus with neutral cues or contexts (for recent reviews, see Herry and Johansen 2014; Tovote et al. 2015) and subsequently express fear responses to those.

BOX 1. Footshock-associated pain as informative threat stimulus supporting fear learning

Despite being the most widely used stimulus to study fear learning, whether a nociceptive event per se induces innate fear is controversial. Typically, innate fear responses to natural threats allow animals to avoid harm without having to experience it (Blanchard and Blanchard 1988). Pain, on the other hand, is a more universal signal of harm that presumably developed to help animals avoid stimuli that are not registered by their innate fear systems. If this is true, then pain (despite being innately aversive) should not induce innate fear, but instead should act as an informative unconditioned stimulus (US) to drive conditioned responses (CR) associated with the pain context or cue (which serves as the conditioned stimulus, or CS). Evidence for this interpretation can be found in the immediate shock deficit. Several experiments have shown that rodents do not show defensive behaviors like freezing, passive avoidance, or potentiated startle when the shock is delivered in a novel context without delay. This, in turn, suggests that defensive responses observed when rodents receive a shock after they have been exposed to a context for a certain time frame are most likely conditioned responses to such context and not innate responses to the footshock (Bolles and Fanselow 1980; Fanselow 1990; Kiernan et al. 1995; Landeira-Fernandez et al. 2006).

The neural processes underlying this form of emotional learning—and behavioral tests to elicit this response—are by far the most widely used to study conditioned fear, and the circuits governing the encoding, retrieval, and extinction of such responses are consequently well described (for recent reviews, see Herry and Johansen 2014; Tovote et al. 2015). In brief, this foot-shock US–CS associative fear learning system is centered in the basolateral amygdalar complex (BLA), which integrates unconditioned and conditioned signals and harbors plasticity mechanisms that form the basis of fear memory encoding. In particular, CS information is directly targeted to the BLA complex through auditory and somatosensory thalamic and cortical inputs, whereas US signals to the BLA may arise from indirect inputs from the PAG or other cortical areas (Fig. 2; Johansen et al. 2010, Herry and Johansen 2014). The BLA complex then projects to the CEA, a structure necessary and sufficient for the initiation of various defensive responses via projections to multiple downstream targets (LeDoux 2003), including the vlPAG, which mediates freezing and behavioral inhibition (LeDoux et al. 1988), and the substantia innominata, which mediates arousal, attention, and active behaviors (Gozzi et al. 2010). In addition, the CEA projects to the lateral and dorsal hypothalamus facilitating the hypothalamus-pituitary-adrenal gland (HPA) axis activation (LeDoux 2003; LeDoux et al. 1988) and to the dorsal motor nucleus of the vagus nerve and parabrachial nucleus (Bandler and Shipley 1994; Hopkins and Holstege 1978) mediating parasympathetic autonomic responses.

Importantly, a growing body of evidence indicates that the CEA not only serves as a output structure of the BLA, but also harbors plasticity mechanisms necessary for fear learning itself (Keifer et al. 2015; Wilensky et al. 2006; Sato et al. 2015; Ciocchi et al. 2010; Duvarci et al. 2011). In particular, a recent study has shown that the CEA directly integrates US nociceptive signals from the parabrachial nucleus and CS stimuli deriving from the auditory thalamus via the LA (Han et al. 2015).

The neural circuits at the basis of this type of emotional learning are centered in the basolateral amygdalar complex (BLA) and have been studied in great detail (Box 1). Yet, how nociceptive signals are detected by the BLA learning system is still unclear. Some studies suggest that the sensory discriminative component of the nociceptive information is conveyed to the BLA via a spino-thalamic tract composed of lamina I spinal cord neurons targeting the posterior intralaminar thalamic nucleus (PIN), which in turn sends excitatory projections to the BLA (Asede et al. 2015; Bienvenu et al. 2015). However, this hypothesis has been challenged by studies showing that fiber-sparing lesions in this area do not impair footshock learning (Brunzell and Kim 2001; Lanuza et al. 2008; Herry and Johansen 2014). An alternative input carrying nociceptive information to the BLA might originate from the PAG through midline thalamic or other cortical relays, because the PAG integrates nociceptive inputs from the spinal and trigeminal dorsal horn (Johansen et al. 2010) and PAG electrical stimulation is sufficient to instruct BLA-dependent fear learning (Di Scala et al. 1987; Kim et al. 2013). Similarly, pharmacological inhibition of PAG during fear conditioning impairs footshock-induced fear memory encoding (Johansen et al. 2010).

Furthermore, nociceptive information is also directly detected by the CEA, a structure serving as the main output of BLA-mediated conditioned fear responses (Box 1). This amygdalar sub-region receives nociceptive signals from the parabrachial nucleus (PB, Bernard and Besson 1990), responds to nociceptive stimuli (Neugebauer and Li 2002) and its inactivation impairs fear learning (Wilensky et al. 2006). Moreover, recent studies have nicely identified a putative circuit mediating CS–US associative learning centered in the CEA, composed of spino-parabrachial afferents conveying the affective component of nociceptive information to the CEA (Han et al. 2015; Sato et al. 2015; Sugimura et al. 2016). Taken together this evidence suggests that both the BLA complex and the CEA contribute to footshock fear memory encoding; however, the interplay within these two structures in the processing of footshock fear learning remains poorly understood.

### Suffocation signals

The neural circuits of fear induced by suffocation signals have attracted particular attention for their relevance to panic disorder, where hyper-responsivity of this system has been proposed to represent a major contributor to this pathology (Klein 1993; Preter and Klein 2008; 2014). Obstructions to respiration invoke intense innate responses across animal species (Schimitel et al. 2012). The brain is thought to detect suffocation through sensors of blood O_2_ and CO_2_ partial pressure located in the carotid body (Finley and Katz 1992). Hypoxia (low O_2_) or hypercapnia (high CO_2_) signals from the carotid are processed by the nucleus of the solitary tract (NTS) that, in turn, targets respiration nuclei in the medulla for respiratory adaptations (Loewy and Burton 1978; Paton et al. 2001), as well as higher structures involved in the generation of defensive responses such as PAG, CEA, and the paraventricular nucleus of the hypothalamus (Ricardo and Tongju Koh 1978). Indeed, severe hypoxia induces significant increases in c-Fos protein expression in the NTS and in the dorsolateral and lateral columns of the PAG (Casanova et al. 2013), and lesions in the PAG suppress hypoxia-induced defensive behaviors (Schimitel et al. 2012). Moreover, slice electrophysiology experiments have shown that the PAGd harbors hypoxia-responding neurons (Kramer et al. 1999). Taken together, these findings indicate that the dorsal PAG may serve as a central node in the processing of hypoxia-induced fear (Schenberg et al. 2014).

## Integration unit

Once the brain detects a threat, the associated information is conveyed from primary sensory structures to downstream structures. There, all the sensory information coming from the threat, the environment, and the subject's internal state are integrated to recruit—in an orchestrated manner—the downstream effectors initiating the most appropriate motor and homeostatic responses. The brain circuits occupying such an intermediate position in the sensory-motor processing of fear responses constitute what we define here as an “integration functional unit.” Originally, it has been hypothesized that this fear integration circuit consists of a unique set of nuclei centered in the amygdala that processes signals from all types of threats to give rise to a stereotyped set of defensive responses (Bolles and Fanselow 1980; Fanselow 1994). However, this view has been challenged by a number of subsequent studies, indicating that the brain processing of innate fear occurs in separate circuits depending on the type of threat. Such high degree of functional segregation is not only observable at the level of the detection unit, but also of downstream integration functional elements.

In particular, in contrast to the amygdala-centered footshock fear circuit, conspecific and predator fear are primarily integrated by parallel circuits located in the medial hypothalamus (Fig 1; Gross and Canteras 2012; Silva et al. 2013). These hypothalamic circuits, despite being ethologically relevant, have received less attention compared to the deeply investigated amygdalar fear circuits. In the following section we summarize the organization of the functional integration unit processing predator, conspecific and footshock fear, with a particular focus on recent studies highlighting the hypothalamic function in fear integration.

### The predator fear circuit

In rodents the core integration unit for fear to predatory threats resides in the medial hypothalamic defensive system (MHDS; Canteras 2002). This system consists of a set of nuclei located in the hypothalamic medial zone: the anterior hypothalamic nucleus (AH), the dorsomedial portion of the ventromedial hypothalamus (VMHdm), and the dorsal premammillary nucleus (PMD). These three highly interconnected nuclei are selectively recruited by predator exposure (and not by conspecific threat or pain), receive inputs from sensory circuits detecting predatory cues, and target defense output structures like the PAG (Canteras 2002)*.* Accordingly, inhibition of these nuclei impairs defensive responses to predators while their artificial activation promotes defensive responses in both rodents and primates (Lipp and Hunsperger 1978; Canteras et al. 1997; Blanchard et al. 2005; Wilent et al. 2010; Pavesi et al. 2011; Silva et al. 2013; Kunwar et al. 2015; Wang et al. 2015).

The functional anatomy of the medial hypothalamic defensive circuit mediating predator fear has been described in detail (for reviews, see Canteras 2002; Gross and Canteras 2012). It receives massive inputs from sensory structures of the detection units through two amygdalar regions: the medial nucleus (MEA), which conveys olfactory information from the vomeronasal organ; and the basomedial nucleus (BMA), which conveys wider polymodal information about the predatory threat through its BLA inputs, which in turn receives information from olfactory, insular, and prefrontal cortices. Importantly, parallel amygdalar inputs are also relayed to the MHDS by the interfascicular nucleus of the bed nucleus of the stria terminalis, a structure implicated in aversion processing (Walker et al. 2003). In addition, the MHDS is also targeted by the hippocampal–septal system, conveying processed sensory information including spatial and novelty signals (Canteras 2002). Although never directly tested, information conveyed by these inputs may be important to best adapt the behavioral output strategy to contextual features such as availability of an escape route and proximity of the threat. In addition, the MHDS also receives afferents from the parabrachial nucleus (Bester et al. 1997), a structure detecting the bodily internal state, and from the medial prefrontal cortex, probably exerting a top-down control on defensive responses (Comoli et al. 2000).

The major outputs of the MHDS target motor output initiators such as the PAGd and SC and thereby mediate behavioral defensive responses to predators. In addition, the MHDS also regulates autonomic and endocrine functions through efferents to the dorsomedial hypothalamic nucleus, the vagal motor nerve, and the paraventricular hypothalamic nucleus (Canteras 2002).

All this evidence indicates the fundamental role of the MHDS in integrating signals from the predatory threat detection unit and in orchestrating responses targeting the output unit. Nevertheless, it is important to note that even if the MHDS plays a central role in integrating predator fear, anti-predator defense can, in some specific cases, rely on alternative circuits bypassing the hypothalamus (Kunwar et al. 2015). For instance, rapid responses to looming stimuli seem to rely on more direct inputs to PAG from SC (Zhao et al. 2014; Shang et al. 2015) and ultrasound-induce defensive responses may rely mainly on circuits centered in the IC (Xiong et al. 2015). What is more, recent studies have argued that the MHDS is not only involved in the sensory-motor processing of defensive responses but, at the same time, also actively serves to encode a generalized internal motivational state of fear that may reflect the emotion associated with threat exposure (see below; Kunwar et al. 2015; Silva et al. 2016). Interestingly, some studies showed that electrical stimulations of the VMH, a nucleus of the MHDS, in humans elicited panic attacks (Wilent et al. 2010), pointing to a possible conservation of this predator fear integrator also in humans.

### The conspecific fear circuit

Surprisingly, rodents show escape and defensive responses to an aggressive conspecific display activation of a set of brain structures that do not overlap with the ones recruited by exposure to a predatory threat or a physically harmful stimulus (Motta et al. 2009; Silva et al. 2013). The core integration unit processing conspecific fear overlaps with the medial hypothalamic reproductive system known to be recruited during sexual and aggressive behavior. This set of nuclei includes the medial preoptic nucleus (MPN), ventrolateral portion of the ventromedial hypothalamus (VMHvl), the ventral premammillary nucleus (PMV) and dorsomedial portion of the dorsal premammillary nucleus (PMDdm) (Kollack-Walker and Newman 1995; Kollack-Walker et al. 1999; Motta et al. 2009; Lin et al. 2011; Yang et al. 2013). Accordingly, pharmacogenetic inhibition of the VMHvl (Silva et al. 2013) or lesions of the PMDdm decreases defensive responses to an aggressive conspecific (Motta et al. 2009), indicating that this hypothalamic system is necessary to process social fear. Importantly, selective inhibition of the nearby MHDS does not alter defensive responses to conspecific aggressor demonstrating a double dissociation between these two medial hypothalamic defense systems (Silva et al. 2013). Like the MHDS, the medial hypothalamic reproductive system receives inputs from the MEA (although from a different sub-region to the one processing predatory cues) conveying pheromone signals from the AOS, and projects to motor output system centered in the PAGd (Canteras et al. 1994; 1995; Motta et al. 2009).

The overlap between the conspecific defense and reproductive and aggressive medial hypothalamic systems is intriguing. Recent insights about the relation between sex and aggression-regulating neurons in the VMH indicate that largely nonoverlapping sets of neurons in the VMHvl may mediate mounting and attack (Lin et al. 2011; Yang et al. 2013). However, how these populations relate to the cells mediating conspecific defensive responses remains to be determined. Likewise, a better understanding of the co-processing of conspecific defense and aggression in the medial hypothalamus is required to determine how animals select or switch behavioral strategies during agonistic encounters.

### The footshock fear circuit

As discussed above (Box 1), painful stimuli are not considered to elicit innate defensive responses, but instead to serve as informative unconditioned stimuli that instruct associative learning to environmental cues. Because of the methodological advantages of studying conditioned responses in the laboratory, the neural circuits supporting footshock-induced conditioning have been extensively investigated (for reviews, see Herry and Johansen 2014; Tovote et al. 2015). This neural integration is mediated by a dedicated amygdala pathway (Box 1, Fig. 2C) that does not seem to overlap with conspecific or social fear processing structures (Gross and Canteras 2012).

Importantly, pain signals often accompany the encounter with other classes of threats such as aggressive conspecifics or predators. However, the integration of noxious stimuli in the predator and conspecific fear systems has thus far no been investigated. One possible source of cross talk between pain signals and predator and conspecific fear systems may originate from the parabrachial nucleus, a structure integrating visceroceptive pain and targeting both the CEA and the medial hypothalamic defensive circuit (Bernard and Besson 1990; Bester et al. 1997), but this remains speculation at this point.

### Internal states—emotional states

The study of fear as an emotion in animals has been challenged because it remains difficult to test whether animals are experiencing a conscious feeling of emotion analogous to that of humans (Ledoux 2012, 2014). Nevertheless, specific criteria have recently been proposed for identifying the neural correlates of an internal state with emotion-like value (Anderson and Adolphs 2014). A brain structure mediating such an internal emotional state, first, should integrate signals from different sensory modalities including innate and conditioned stimuli; second, should be necessary and sufficient to drive a diverse set of environmentally appropriate defensive responses; and, third, its neural activity should be required to instruct a memory of the experience. The fulfillment of these criteria implies that a structure is not merely a relay station of a primary sensory input, nor an initiator of a specific motor response, but, instead, occupies an intermediate position giving rise to an internal state with an a possible human-like emotional value (Anderson and Adolphs 2014). A very limited number of studies have investigated the function of innate fear integration structures in this perspective. The few that have suggest that, at least in the case of predator fear, the medial hypothalamic defensive system may serve as a central processor of an internal emotional state. In the following section, we summarize these recent findings. In the future, similar studies need to be conducted to explore possible homologous structures mediating an internal state of fear induced by other types of threats such as social or nociceptive ones.

#### MHDS, integration of diverse sensory stimuli

A number of tract-tracing studies have shown that the MHDS receives projections from a wide range of sensory processing areas (Canteras 2002). For instance, the MHDS receives vomeronasal information from the medial amygdala (mainly targeting the VMH), but also from the BMAp that, in turn, receives input from the BLA. The MHDS is therefore well positioned to integrate information from olfactory, insular, and prefrontal cortical areas. The MHDS also receives information about novelty and context from the hippocampus via inputs from the septum to AH (Canteras 2002). And finally the MHDS receives projections from the PB, an important relay area of noxious information (Canteras 2002). The diversity of inputs to the MHDS makes it an ideal candidate structure able to respond to a wide variety of sensory information about environmental threats. Accordingly, loss-of-function studies confirm that selective impairment of VMHdm neurons not only reduces defensive responses to a predator (Silva et al. 2013) but also reduces tone fear conditioning and anxiety (Kunwar et al. 2015), as well as defensive responses to a context previously associated with a predator (Silva et al. 2016).

#### MHDS, induction of multiple defensive behaviors

Both loss- and gain-of-function studies indicate that the MHDS is able to support a variety of environmentally appropriate defensive behaviors and is not bound to a specific behavioral or physiological response. For example, blockade of the VMHdm or PMD impairs freezing, avoidance, escape, risk assessment, anxiety, and autonomic responses (Blanchard et al. 2003, 2005; Silva et al. 2013; Cheung et al. 2015; Kunwar et al. 2015), while optogenetic activation of the VMHdm induces flights, freezing, autonomic activation, and the interruption of ongoing behaviors (Lin et al. 2011; Kunwar et al. 2015; Wang et al. 2015). Interestingly, VMH activation seems to evoke defensive behaviors in a scalable fashion, with less intense stimulation inducing freezing and more intense stimulation evoking activity bursts (Kunwar et al. 2015). Different VMH outputs may regulate different defensive behaviors, with projections to the PAGd mediating freezing and projections to the AH mediating risk assessment and flight (Wang et al. 2015), although both outputs must presumably eventually reach the PAG to produce defensive responses. The VMH also activates sympathetic nervous system responses including pupil dilation, tachycardia, and hyperventilation (Satoh et al. 1999; Wang et al. 2015), which may be mediated by its direct projections to the rostral ventrolateral medulla (RVLM) (Lindberg et al. 2013) a structure harboring catecholaminergic neurons and capable of mediating sympathetic outflow (Burke et al. 2011).

#### MHDS, role in memory formation

Importantly, the MHDS is necessary and sufficient for mediating both innate and learned defensive responses. Lesions and pharmacological inhibition of the PMD and VMHdm reduce acute responses to a predator, but also inhibit defensive responses to the context where the predator was encountered at a later time point (Cezario et al. 2008; Do-Monte et al. 2008; Silva et al. 2013; Silva et al. 2016). Similarly, pharmacological activation of β-adrenergic receptors within this structure is sufficient to serve as an unconditioned stimulus to drive contextual fear learning (Pavesi et al. 2011) and optogenetic stimulation of VMHdm is sufficient to instruct learning in a conditioned place aversion test (Kunwar et al. 2015). Taken together, these results indicate that the MHDS is necessary and sufficient for the encoding and recall of fear memory.

## Fear response output unit

### Defensive responses in rodents

When animals are exposed to a threat, they activate a range of immediate and delayed responses aimed at coping with it. Such responses are adapted to the type of threat and to the circumstances under which the threat is presented. For example, flight is preferred over freezing if an escape route is available and these responses show scalability depending on the proximity of the threat (Fanselow and Lester 1988; Blanchard and Blanchard 1990). In contrast, fear responses to ambiguous threat cues, like predator odor or open and bright spaces typically elicit risk assessment responses, including careful scanning of the environment in a crouched position (crouched sniffing) and attempts to approach the threatening stimulus by stretching the body (stretch postures) followed by rapid escapes to a shelter if available (Blanchard and Blanchard 1988). Moreover, fear responses tend to inhibit other types of ongoing motivated behaviors like feeding and mating (Blanchard and Blanchard 1988). This complex array of defensive behaviors, together with the endocrine and autonomic adjustments occurring upon threat exposure, is orchestrated by specific fear response output circuits.

### PAG: a common output for defense

In their seminal study Hunsperger et al. discovered that fear responses elicited by stimulation of the amygdala or the hypothalamus could be reversed by PAG lesions, but not the other way around (Hunsperger et al. 1963). Since then, a growing body of evidence has accumulated pointing to the PAG as the final common path for all types of defensive responses. First, neuronal activation in the PAG has been observed upon exposure to a wide variety of threats, including live predator or predator odor (Cezario et al. 2008), aggressive conspecifics (Motta et al. 2009), loud ultrasound stimuli (Mongeau et al. 2003), and electrical footshock (Johansen et al. 2010). Second, impairment of PAG function diminishes the expression of defensive behaviors including freezing, risk assessment, flight, analgesia, and autonomical arousal (Hunsperger et al. 1963, Schenberg et al. 2014; Sukikara et al. 2006; Silva et al. 2013). Third, stimulation of the PAG is sufficient to induce defensive responses of various types, including freezing, escape, and jumping (Bandler and Shipley 1994; Schenberg et al. 2014).

The PAG is divided into rostro-caudal columns that have been proposed to control different behavioral outputs (Bittencourt et al. 2004). The dorsal columns of the PAG (PAGd) represent the main output structures of the medial hypothalamic defensive system (Canteras et al. 1994; Canteras 2002; Gross and Canteras 2012) as well as of the SC and IC (Canteras et al. 1992) and mediate active defensive responses to imminent threats like predators and aggressive conspecifics (Sukikara et al. 2006; Motta et al. 2009; Silva et al. 2013). Such active defensive strategies include escape, freezing associated with muscular tension, tachycardia, hypertension, hypervigilance, and hyperreactivity (Schenberg et al. 2014; Sukikara et al. 2006; Wang et al. 2015). The dorsal columns of the PAG project to downstream brain stem structures, including the cuneiform nucleus, periabducens region of the rostral dorsomedial pons, the locus coeruleus, and the ventromedial, ventrolateral, and dorsal medulla (Bandler and Shipley 1994). However, still little is known about how these diverse outputs may differentially contribute to the generation of the wide variety of PAGd-mediated defensive responses.

In contrast, the ventral PAG columns (PAGvl) receive strong inputs from CEA and appear to mediate passive defensive responses associated with parasympathetic activation (bradycardia, hypotension, and hyporeactivity), antinociception, and vocalization (Fanselow 1991; Bandler and Shipley 1994; LeDoux 2003; Johansen et al. 2011), a defensive strategy typically observed in rodents exposed to neutral cues previously associated with footshock. The PAGvl seems to mediate these passive defensive responses though its downstream projections to the ventromedial and ventrolateral medulla and to the cerebellum (Vianna and Brandão 2003; Koutsikou et al. 2014; Tovote et al. 2016). However, here too, the precise contribution of each target structure to the different behavioral outcomes remains to be determined.

### Environment-appropriate fear responses

A limited number of studies have examined how similar threat stimuli can elicit different defensive responses depending on the context. The CEA seems to contribute to this process as projections to cholinergic forebrain nuclei and PAGvl mediate the switch between active and passive fear responses, respectively (Gozzi et al. 2010). Nevertheless, how information about the environment, like the availability of an escape route, is integrated to switch to the most appropriate defensive strategy remains unclear. Mongeau et al. (2003) showed that mice display a tendency for active or passive defense to the same innate fearful stimulus depending on the levels of stress prior to exposure and that this is reflected in differential c-Fos activation patterns. In particular, mice performing active fear responses showed preferential recruitment of a cortical–amygdalo–striatal circuit, whereas mice showing passive defensive responses displayed higher levels of activation in the ventral lateral septum and periventricular zone of the hypothalamus. Moreover, in a recent study Tovote et al. (2016) showed that the same population of GABAergic neurons in the PAGvl is inhibited by long-range inhibitory CEA neurons promoting freezing responses and activated by flight promoting excitatory neurons located in the PAGd. This suggests that PAGvl GABAergic neurons may serve as a major integrator of different upstream inputs for the appropriate switch between active or passive defensive strategies.

### Fear memorization: how do innate fear circuits instruct memory formation?

Exposure to a threat not only triggers acute responses aimed at immediate coping with the danger, but also brain plasticity that allows the subject to adapt its response to future encounters with similar threats. In this perspective, innate fear circuits must recruit both behavioral output structures and brain areas devoted to memory formation. A number of studies have investigated Pavlovian associative learning that accompany exposure to footshock and have nicely unraveled the circuitry, cell-type contribution, and cellular and molecular mechanisms at the basis of this process (the full description of which is beyond the aim of this review; Herry and Johansen 2014; Tovote et al. 2015).

In contrast, much less is known about the learning processes and plasticity mechanisms at the basis of the memorization of innate fear of more ethologically relevant threats such as predators or aggressive conspecifics. Fear memory upon exposure to these threats has been observed, because rodents show defensive responses to a context previously associated to predatory or social threats and loss-of-function studies have led to the identification of a core learning unit necessary for this memorization process. Unlike acute fear, fear memory seems to rely on this common pathway independently of the type of threat. It involves the hippocampus and amygdala as well as cortical circuits centered on the anterior cingulate, medial prefrontal, retrosplenial, and postrhinal areas (Ledoux 2000; Maren 2001, 2011). Corroborating this hypothesis, pharmacological inhibition or lesioning of these structures impairs memory encoding of fear of all types of threats while leaving acute responses unaffected. For example, impairment of BLA function prevents fear learning in animals exposed to footshock (Helmstetter and Bellgowan 1994), predators (Martinez et al. 2011), and aggressive conspecifics (Jasnow and Huhman 2001); similarly, inhibition of the ventral hippocampus impairs fear learning to footshock (Maren 1999), predators (Blanchard et al. 2005), and aggressive conspecifics (Markham et al. 2010).

On the other hand, several studies indicate that integration structures directly driving innate fear are also crucial to generate a memory of the aversive event. Even if the functional interactions of these circuits with the amygdalo–hippocampal–cortical learning unit are not fully understood, one possible model is that innate fear circuits for all types of threats convey threat signals to the higher learning unit where the association with contextual signals may take place (Fig. 2). This hypothesis is yet to be fully demonstrated, but a growing body of studies is providing functional and anatomical evidence for an interplay between the different innate fear circuits and structures devoted to memory formation.

For example, in the case of the predator fear system, both loss- and gain-of-function studies have demonstrated the causal implication of the MHDS in fear learning: Lesions or pharmacological inhibition of the PMD and of the VMHdm before predator exposure impairs fear responses to the environment where the predator was presented (Cezario et al. 2008; Do-Monte et al. 2008; Kunwar et al. 2015; Silva et al. 2016) and their activation is sufficient to induce conditioning (Pavesi et al. 2011; Kunwar et al. 2015). Communication from the MHDS to the cortico-hippocampal learning unit seems to occur through thalamic projections. Specifically, the PMD sends collaterals to MTN including the anteromedial thalamic nucleus (AM, Canteras et al. 2001) which, when blocked before predator exposure, impairs defensive responses to the context previously associated with a predator leaving acute responses to the predator unaffected (Carvalho-Netto et al. 2010; de Lima et al. 2016). The AM projects to the ACC, retrosplenial, entorhinal, perirhinal corteces, and the subiculum (Van Groen et al. 1999), areas implicated in contextual fear learning (Ledoux 2000; Maren 2001, 2011).

A second route of communication from the predator fear circuit to memory systems may stem from the PAGd. Pharmacological activation of PAGd is sufficient to induce defensive responses to the context where the stimulation occurred (Kincheski et al. 2012). The mnemonic effects of PAG stimulation may be mediated by PAG projections to PMD, as well as by direct projections to thalamic nuclei including intralaminar, laterodorsal, reuniens, suprageniculate, and subparafascicular thalamic nuclei (Kincheski et al. 2012). However, pharmacogenetic inhibition of dorsal PAG does not impair predator learning despite reducing acute fear responses (Silva et al. 2016), implying that at least for VMH, the PAG does not mediate the mnemonic effects of MHDS stimulation, but by a so far unknown route.

Similarly to predator fear, conspecific fear memory encoding requires the BLA, prefrontal cortex, and the ventral hippocampus (Markham et al. 2010; 2012). However, it is less clear how the conspecific fear circuit instructs the fear learning unit. Similar to the predator fear circuit, the dorsomedial PMD, a subregion of the PMD mediating acute conspecific fear (Motta et al. 2009), might contribute to instruct fear memory through projections to MTN that, in turn project to cortical and hippocampal structures, but this remains highly speculative.

Painful stimuli such as an electrical footshock also have the capability to instruct memory formation via a learning system relying on the amygdala, hippocampus, and prefrontal cortex (see “Box 1,” “pain as an instructive threat stimulus,” and “the footshock fear system”) and a plethora of studies has investigated this memory process (for recent reviews, see Herry and Johansen 2014; Tovote et al. 2015). Interestingly, recent studies have started to investigate how neurons responding to innate fear stimuli interact with neurons mediating learned responses to a neutral cue. For instance, a recent study nicely showed that a specific neuronal population in the CEA mediating innate fear to predator odor inhibits learned freezing responses to a foot-shock associated cue. This finding indicates that this neuronal population in the CEA serves as a hierarchy generator between defensive responses that prioritizes innate fear over learned fear responses (Isosaka et al. 2015). In the same perspective another recent study showed that neurons in the BLA selectively mediate conditioned responses to a neutral stimulus by recruiting innate fear neurons to generate defensive responses (Gore et al. 2015).

It is important to bear in mind that many aspects of predator and social fear memory remain unknown. First, to our knowledge, no study has investigated the neuronal plasticity and molecular mechanisms underlying this form of memory. Second, the brain structures and cell types harboring these changes have yet to be identified. And, third, it remains unclear if fear memory following exposure of social and predatory threats is truly a pure associative type of learning, because also nonassociative components like generalized hyperarousal have been described (Fifield et al. 2015).

## Conclusions and future perspectives

Here we aimed to summarize in a comprehensive manner how the brain processes threat signals to generate both acute and long-term innate fear responses. In particular, we were interested in framing together fear systems recruited by a vast variety of threats. We propose that it is conceptually useful to divide fear-processing structures in the brain in three fundamental functional units: a detection unit, an integration unit, and an output unit, which interact with a common memorization system. Moreover, we outline that the processing of acute fear resides in separate circuits at all functional levels depending on the type of threat, whereas they seem to recruit the same brain systems to instruct a memory of the fearful event.

Importantly, these different fear systems have only been investigated in separate studies, leaving unclear how they interact with each other. To address this issue, more comprehensive studies investigating the different fear systems in a unitary fashion will be required. Moreover, the interplay between the innate and learned fear systems remains poorly understood. For example, a recent study showed that CEA neurons mediating innate fear responses to predator odor can serve as a hierarchy generator prioritizing innate fear responses over learned ones (Isosaka et al. 2015)

Another important issue is that the investigation of the neural correlates of fear in rodents have classically been limited to the sensory-motor processing of defensive responses to a threat and only few studies have tried to uncover the brain circuits generating an “emotional state of fear” (Kunwar et al. 2015; Silva et al. 2016). Taking into consideration the translational potential of rodent studies to understand human physiology, this would be a crucial point as it would pave the way to understand how the circuits identified in the mouse relate to the brain correlates of human emotion of fear. In the future we should thus include these criteria in the study of the neural correlates of fear in rodents and aim beyond the classical sensory motor model of defensive responses.

Along these lines, a number of fMRI studies investigating the neural correlates of fear in human subjects have found that amygdalar activity is associated with fear states (LaBar et al. 1998). However, other fear systems identified in rodents are also related to fear states in humans. In an fMRI study in humans, Mobbs et al. (2007) showed that as a virtual predator approached the subjects, brain activity shifted from the ventromedial prefrontal cortex to the PAG. In addition, electrical stimulation at the level of the PAG and VMH in human subjects induced panic and the sensation of being chased, respectively (Amano et al. 1982; Wilent et al. 2010), suggesting that fear circuits may rely on similar structures as in rodents. Moreover, a recent study showed that patients with bilateral amygdala lesions developed panic attacks upon exposure to a CO_2_ inhalation test, which suggests that an alternative pathway mediates panic in humans (Feinstein et al. 2013). These findings indicate that the functional segregation of different kinds of fear may be conserved in humans, which is important to consider in the study of the human pathophysiology of fear.

Accordingly, particular attention should be paid to investigate how the different fear circuits described above may be dysregulated in fear-related pathological states like phobias, post-traumatic stress, and anxiety disorders. For example, it has been hypothesized that panic disorder resides in a dysregulation of the suffocation alarm system (Klein 1993; Preter and Klein 2008; 2014), but clear experimental evidence for this is lacking. In extension, it would also be interesting to assess how genetic predisposition for these pathological states might alter not only learned fear responses, but also innate fear responses, and whether an excessive innate fear response might predispose an individual for exaggerated learned fear responses.

Lastly, it is important to note that most of our knowledge about the functional dissociation of different brain circuits arises from older studies, in which brain function was inferred using a combination of anatomical tract-tracing studies, c-Fos-based functional mapping, lesions, and pharmacological inhibition. Although these studies provided fundamental insight in the function of fear circuits, they have major limitations such as temporal and cellular resolution. The advent of new tools for the investigation and manipulation of genetically defined populations of neurons such as optogenetics, chemogenetics, endoscopic calcium imaging, together with novel genetically encoded neuro-anatomical tracing tools are likely to yield major breakthroughs in the field and will allow answering these and other open questions at better spatiotemporal resolution in future studies.
